# Gut Leakage Markers and Cognitive Functions in Patients with Attention-Deficit/Hyperactivity Disorder

**DOI:** 10.3390/children10030513

**Published:** 2023-03-05

**Authors:** Sheng-Yu Lee, Sung-Chou Li, Chia-Yu Yang, Ho-Chang Kuo, Wen-Jiun Chou, Liang-Jen Wang

**Affiliations:** 1 Department of Psychiatry, Kaohsiung Veterans General Hospital, Kaohsiung 813, Taiwan; 2 Department of Psychiatry, College of Medicine, Kaohsiung Medical University, Kaohsiung 807378, Taiwan; 3 Department of Medical Education and Research, Kaohsiung Veterans General Hospital, Kaohsiung 813, Taiwan; 4 Department of Microbiology and Immunology/Molecular Medicine Research Center, Chang Gung University, Taoyuan 333, Taiwan; 5 Department of Otolaryngology Head and Neck Surgery, Chang Gung Memorial Hospital, Taoyuan 333423, Taiwan; 6 Department of Pediatrics, Kaohsiung Chang Gung Memorial Hospital, Chang Gung University College of Medicine, Kaohsiung 83301, Taiwan; 7 Kawasaki Disease Center, Kaohsiung Chang Gung Memorial Hospital, Kaohsiung 83301, Taiwan; 8 Department of Child and Adolescent Psychiatry, Kaohsiung Chang Gung Memorial Hospital, Chang Gung University College of Medicine, Kaohsiung 83301, Taiwan

**Keywords:** ADHD, biomarkers, gut leakage, cognitive function, neurodevelopment

## Abstract

Attention-deficit/hyperactivity disorder (ADHD) is a commonly seen mental disorder in children. Intestinal permeability may be associated with the pathogenesis of ADHD. The study herein investigated the role of gut leakage biomarkers in the susceptibility of ADHD. A total of 130 children with ADHD and 73 healthy controls (HC) individuals were recruited. Serum concentrations of zonulin, occludin, and defensin (DEFA1) were determined. Visual attention was assessed with Conners’ continuous performance test (CPT). In order to rate participants’ ADHD core symptoms at home and school, their parents and teachers completed the Swanson, Nolan, and Pelham—Version IV Scale (SNAP-IV), respectively. We found significantly lower DEFA1 levels in the ADHD group compared to that in the HC group (*p* = 0.008), but not serum levels of zonulin and occludin. The serum levels of DEFA1 showed an inverse correlation with the inattention scores in the SNAP-IV parent form (*p* = 0.042) and teacher form (*p* = 0.010), and the hyperactivity/impulsivity scores in the SNAP-IV teacher form (*p* = 0.014). The serum levels of occludin showed a positive correlation with the subtest of detectability in the CPT (*p* = 0.020). Our study provides new reference into the relation between gut leakage markers and cognition, which may advance research of the pathophysiology of ADHD.

## 1. Introduction

Attention-deficit/hyperactivity disorder (ADHD) is a neurodevelopmental disorder commonly seen in children and may persist into adulthood [1]. Interventions early in life are important to improve quality of life and help achieve effective strategies to deal with the functional impact of ADHD symptoms. However, pharmacological or nonpharmacological interventions may not induce sustained remission of symptoms or cure this disorder. According to recent research, the recognition and diagnosis of ADHD are still relatively limited in most countries, especially in girls [2]. If it persists into adulthood, ADHD may increase the risk for other psychiatric comorbidities and adverse outcomes such as academic underachievement, socio-occupational struggling or even criminality [2]. Increasing numbers of studies have even reported a heightened risk of accidents due to unnatural causes, unintentional injuries, and susceptibility to contracting COVID-19 in patients with ADHD [3,4,5]. Besides clinical symptoms, children with ADHD are also found with emotional dysregulation, worse performance in multiple cognitive domains including alerting and executive attention and executive skills compared to those without ADHD [6,7].

Multiple factors are involved in the etiology of ADHD, including genetic, environmental and psychosocial factors. Recently, the link between intestinal function, gut microbiota and the central nervous system, referred to as dysfunctionality of the “gut–brain axis”, has also been of interest contributing to the underlying pathophysiology of ADHD [8,9,10]. The communication pathway between the gut and the central nervous system was suggested to play a role in the regulation of immune system, mood and cognition. Therefore, increased intestinal permeability, also known as “leaky gut”, has gained attention as a candidate associated with the pathophysiology of mental disorders including ADHD. It was reported that microbiota may use cytotoxins or enterotoxins in the intestine to infiltrate the gut epithelial cells and alter the interaction of cytoskeletal proteins [11]. With increased intestinal permeability, increased intestinal bacteria pass into the blood then to the brain and further trigger neuroinflammation response, contributing to the etiology of ADHD [12]. Epithelial tight junction defensins are important for the intestinal barrier and have, therefore, been proposed as potential markers of mucosal permeability, including zonulin, occludin, and Defensin alpha 1 (DEFA1) [13,14]. Zonulin modulates not only gut–brain barriers but also blood–brain barriers, as it is the principal regulator of endothelial and epithelium tight junctions [15,16,17]. Zonulin is involved in balancing the immune response. The upregulation of zonulin may increase the permeability of macromolecules and thus lead to the uncontrolled inflow of infectious antigens. Zonulin has been proposed as playing a role in the pathogenesis of some chronic inflammatory disorders, such as diabetes type 1 and 2 [18,19] and obesity [20]. It has also been proposed as a peripheral marker for the above metabolic disease. Elevation of zonulin levels have been linked with a worsening in the manifestations of hyperactivity and impairment in social functioning in children with ADHD [12]. Occludin, part of the tetraspan family of integral membrane proteins, is an important element of the tight junction which may cause the weakening of the tight junction barrier and increase the penetrability of the gut wall [21]. Besides the gut wall, occludin has been proposed as a major component of the blood–brain barrier (BBB). Occludin was found to control the cohesion and permeability of the BBB. Furthermore, the level of expression of occludin protein in the endothelial cells of the brain regulate the function of BBB [22]. In this way, occludin has been proposed as a predictor to evaluate BBB damage in patients suffering from acute ischemic stroke [23]. Defensin alpha 1 (DEFA1) belongs to a family of peptides which function as host defenses through the mechanisms of being toxic to cells and bacteria [24]. DEFA1 is abundant in the neutrophils and may assist with phagocyte-mediated host defense. DEFA1 is frequently found in the epithelia of mucosal surfaces including the intestine. DEFA1 may manifest proinflammatory activities in the intestine by triggering the release of interleukin-6 (IL-6) in macrophages and boosting the local inflammatory response in the gut; these activities amplify systemic inflammation and subsequently lead to intestinal leakage [25,26].

The intestinal microbiota may affect the growth and development of the overall body and multiple organs including the brain [27,28]. With immature and still-unstable gut microbiota, children’s brains may be vulnerable to pathological insult [29]. The blood–brain barrier (BBB) and the neurovascular system may be altered by peripheral immune cells released by the microbiome [30]. The low-grade systemic inflammation caused by the disruption of the intestinal barrier may affect the brain–gut axis profoundly and subsequently impair cognitive development [31]. In addition, dysbiosis, which is characterized by an unbalanced composition of microbiota, may cause the growth of inflammatory microbes, further jeopardizing gut permeability and further giving rise to systemic inflammation [9]. Systemic inflammation may affect the absorption of iron in the intestine [32], while iron deficiency is critical to brain development in children [33]. In addition, systemic inflammation may trigger the disruption of the BBB. On the other hand, oxidative stress induced by dysbiosis may further influence neuron cells and neurotransmitters which are associated with the pathogenesis of ADHD [34]. A meta-analysis showed that the certain bacterial taxa that are mostly correlated with ADHD are still inconclusive [9,35]. It may be of interest to explore the association between gut permeability and the bacterial taxa of ADHD.

We hypothesized that intestinal permeability may have a role in the pathogenesis of ADHD. Therefore, we plan to explore the serum levels of zonulin, occludin, and DEFA1 in patients with ADHD and controls. In addition, we suggested that increased intestinal permeability is involved in the cognitive dysfunction and clinical symptoms of ADHD. Therefore, our study aimed to investigate the association between the peripheral levels of zonulin, occludin, and DEFA1 and the cognitive function and clinical features of ADHD.

## 2. Material and Methods

### 2.1. Study Participants

All subjects gave their informed consent for inclusion before they participated in the study. The study was conducted in accordance with the Declaration of Helsinki, and the protocol was approved by the Ethics Committee of Kaohsiung Chang Gung Memorial Hospital (IRB No. 201702019A3) on 20 February 2020. Children with ADHD were recruited from the Kaohsiung Chang Gung Children’s Hospital and HCs from the local community. Before taking part in this study, all participants and their parents or guardians fully understood all the terms of the study protocols and wholeheartedly agreed to sign forms to participate in this study.

The inclusion criteria for patients with ADHD were as follows: (i) A confirmed diagnosis made by a senior child psychiatrist through structural interviews implementing the Kiddie-Schedule for Affective Disorder and Schizophrenia-Epidemiological Version (K-SADS-E) [36,37]; (ii) children between the age of 6 and 16 years old; and (iii) children who had never received any ADHD-related medications including methylphenidate, atomoxetine, clonidine, or bupropion. The exclusion criteria for children with ADHD were as follows: (i) children who were diagnosed with other major neuropsychiatric diseases, including intellectual disabilities, autism spectrum disorders (ASD), major depressive disorders (MDD), bipolar disorders, psychotic disorders, or substance use disorders (SUD); (ii) children who were diagnosed with any major physical illnesses including neuroendocrine, epilepsy, or gastrointestinal disorders; (iii) children who had taken any antibiotics, anti-inflammatory drugs, or probiotics one month prior to this study or children who were vegetarian.

The HC group included children between 6 and 16 years old having no diagnosis of ADHD in the same catchment area. HCs were also interviewed under the structural interview of K-SADS-E to confirm that they had none of the aforementioned major neuropsychiatric diseases (intellectual disabilities, ASD, MDD, bipolar disorders, psychotic disorders, or SUD). Individuals with any major physical illnesses during recruitment who had ever received any antibiotics, anti-inflammatory drugs, or probiotics one month prior to the recruitment, or who were vegetarians, were also excluded.

### 2.2. Intestinal Permeability Markers Assessment

It was necessary to store the serum samples, which were obtained between 08:00 and 10:00, under the condition of −80 °C until the laboratory assessment started. Concentrations of zonulin in the serum were measured using commercial ELISA assays according to the manufacturer’s protocols from Immundiagnostik AG (Bensheim, Germany). All study samples and standards were checked in duplicate. The serum occludin levels were measured with the Human Occludin ELISA kit (Biomatik, Wilmington, DE, USA), using the sandwich-enzyme-linked immunosorbent assay method. Serum DEFA1 levels were assessed with the Human DEFA1 PicoKine ELISA Kit.

### 2.3. Clinical and Cognitive Assessments

The psychologist adopted Conners’ CPT [38] and Conners’ Continuous Auditory Test of Attention™ (Conners’ CATA™) to examine the visual and auditory attention of each participant [39]. We adopted these two exams because they encompass both visual and auditory attention, which are the two most frequently impaired attentions in ADHD. Previous evidence has indicated that the CPT and CATA neuropsychological tests provide objective information about attention in ADHD cases [40,41]. The measures used in the analyses are omissions, commissions, and detectability (d’). These exams were performed in a room designed to diminish variability in testing conditions. To measure the ADHD core symptoms at home and school, we asked the participants’ parents and teachers to complete the parent and teacher forms of the Swanson, Nolan, and Pelham—Version IV Scale (SNAP-IV), respectively [42,43,44].

### 2.4. Statistical and Bioinformatics Analysis

The sample size was measured with the software package G-Power 3.1, based on the settings of 80% power, *p* = 0.05, and allocation ratio ADHD/control = 2/1. The sample sizes were estimated to be 39/19 to detect a large effect size (Cohen’s d = 0.8); 96/48 to detect a medium effect size (Cohen’s d = 0.5); and 591/295 to detect a small effect size (Cohen’s d = 0.2). In the current study, we planned to detect at least a medium effect size. Data analyses were performed utilizing the Statistical Package for the Social Sciences Version 16.0 (SPSS Inc., Chicago, IL, USA). The average ± standard deviation or frequency was presented in the form of variables. The analysis for the dichotomous variables such as the sex distribution of children with ADHD and HCs was carried out using a chi-square test. As for repeated variables, the differences in demographics, clinical symptoms, and neuropsychological function between patients and controls were decided using an independent *t*-test. Two-tailed *p* < 0.05 was the significance for statistics.

Finally, we performed a sensitivity test in which we excluded four healthy control subjects with particularly high levels of DEFA1. A linear regression model was performed to examine the effect of ADHD status on DEFA1 levels (outcome variable), and sex (m/f) and BMI were the explanatory variables.

## 3. Results

### 3.1. Demographic Data

We recruited a total of 130 children with ADHD (mean age: 9.2 years old, 78.5% male) and 73 HC individuals (mean age: 9.3 years old, 63% male). Table 1 summarizes the demographic characteristics and clinical measures of the ADHD and HC groups. We noticed a higher male-to-female ratio in the ADHD group; however, such a ratio is consistent with the known ADHD prevalence ratio ranging from 2:1 to 10:1 [45].

### 3.2. Clinical and Cognitive Assessment

As shown in Table 1, there were significant differences in gender between the ADHD group and the HC group. Although there were no differences in age, height, or weight between the ADHD and the HC groups, there were significant differences in BMI between the two groups. The ADHD group had a significantly higher BMI compared to the control group. As for clinical manifestations, significant differences in all the clinical measures of SNAP-IV were found between the ADHD group and the HC group. As for cognitive functions, there were significant differences in all the subtests of the CPT and CATA between the ADHD group and the HC group as well. In other words, the ADHD group showed prominent clinical symptoms and worse cognitive functions in all aspects than the HC group.

### 3.3. Gut Leakage Markers Assessment

**Figure 1:**
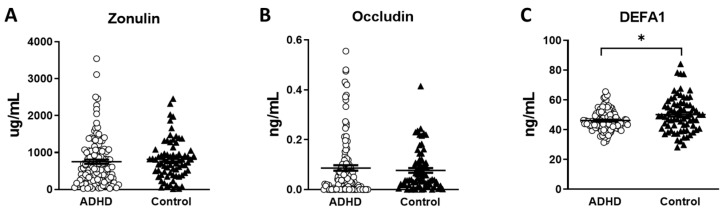
The serum concentration of gut leakage markers in patients with ADHD and healthy controls. (**A**) Zonulin. (**B**) Occludin. (**C**) DEFA1. (○ ADHD group, ⯅  control group, * *p* < 0.05).

The serum levels of the gut leakage markers are shown in Table 1. We found significantly lower DEFA1 levels in the ADHD group than in the HC group (*p* = 0.008) (Figure 1C). The serum levels of zonulin and occludin were not significantly different between the two groups (Figure 1A,B). We further analyzed the correlation of the gut leakage markers with clinical measures and cognitive functions in the ADHD group (Table 2). We found that the serum levels of DEFA1 showed an inverse correlation with the inattention scores in the SNAP-IV parent form (*r* = −0.146 *p* = 0.042) and that in the SNAP-IV teacher form (*r* = −0.187 *p* = 0.010) (Table 2). In addition, the serum levels of DEFA1 also demonstrated an inverse correlation with the hyperactivity/impulsivity scores in the SNAP-IV teacher form (*r* = −0.179 *p* = 0.014) (Table 2). However, we did not find a correlation between the serum levels of DEFA1 with the hyperactivity/impulsivity scores in the SNAP-IV parent form. In addition, the serum levels of zonulin and occludin were not significantly correlated with any of the clinical measures. As for cognitive function, we found the serum levels of occludin showed a positive correlation with the subtest of detectability in the CPT (*r* = 0.166 *p* = 0.020) (Table 2). We did not find correlations between the serum levels of zonulin, occludin, and DEFA1 and other subtests of the CPT or CATA.

### 3.4. Sensitivity Test

Finally, we excluded four healthy control subjects with particularly high levels of DEFA1 (≥77.5 ng/mL). A linear regression model (Table 3) revealed that ADHD status still showed and independent effect on DEFA1 levels (*p* = 0.034), controlling for the potential confounding effects of sex and BMI.

## 4. Discussion

In this study, we found significant differences in the gut leakage marker, DEFA1, between the ADHD group and the HC group. This significant difference remained even after we excluded four healthy control subjects with particularly high levels of DEFA1 and controlling for the potential confounding effects of sex and BMI. The sensitivity test in 3.4 further verified that ADHD status did influence the serum DEFA1 levels. However, we found no significant differences in the other two gut leakage markers, zonulin and occludin, between the ADHD and HC groups. In addition, the serum level of DEFA1 showed an inverse correlation with both the inattention and hyperactivity/impulsivity scores in the SNAP-IV teacher form, demonstrating its inverse correlation with symptoms of ADHD. To our knowledge, our study is the first study to evaluate the association between gut leakage markers and vulnerability, clinical symptoms, and cognitive functions of ADHD.

Our results were different from our initial hypothesis, for we presumed that higher gut leakage markers were associated with the vulnerability of ADHD, and gut leakage markers may positively correlate with clinical symptoms. Our finding of lower DEFA1 levels in the ADHD group compared to controls may not be unsupported. Previous studies found that decreased levels of DEFA1 were associated with several infectious diseases, while increased levels of DEFA1 may protect against the progression of infection [46,47]. DEFA1 is an antimicrobial peptide of the innate immune system. The *DEFA1* gene encodes the human neutrophil peptides. Increased copy numbers of the *DEFA1* gene were correlated with increased levels of the DEFA1 protein [48]. Previous study reported that reduced copy numbers of the *DEFA1* gene was associated with recurrent urinary tract infections in children [49]. It was also reported that lower copy numbers of the *DEFA1* gene contribute to a higher risk for hospital-acquired infections [46]. Another study found that a higher secretion of DEFA1 by immature dendritic cells may protect against the progression of HIV infection [47]. It was proposed that a lower DEFA1 protein level may contribute to impaired innate defenses and a weaker functioning of antimicrobial activity, and consequently leads to infections [46]. Therefore, in the current study, we propose that the significantly decreased DEFA1 level found in the ADHD group may be associated with the fragile functioning of autoimmune and antimicrobial activity. Our result may be another contribution to current findings for we also found an association between lower levels of DEFA1 in ADHD compared to controls, and ADHD is regarded as an inflammatory disease. Our result may be contrary to our previous hypothesis; however, it reminded us that in in vivo studies, levels of certain markers may vary greatly according to clinical condition and a dichotomous hypothesis may not always stand. Previous studies suggested that personal and maternal history of autoimmune disease are associated with a higher risk of ADHD [50]. Although no previous studies ever reported correlations between DEFA1 levels and neither ADHD nor its clinical symptoms (inattention, impulsivity, and hyperactivity), we propose that the inverse correlations we found let us infer that the association between vulnerability to ADHD and the gut leak markers may be connected by infection.

Moreover, we did not find an association between ADHD and other gut leakage markers of zonulin and occludin. Our study agrees with [51] which found no significant difference between ADHD and serum zonulin levels. However, our study did not comply with the study by Ozyurt et al. [12]; the current study sample was comprised of 130 patients with ADHD and 73 controls, which was almost 3 times more compared to the sample size in the Ozyurt study. The differences in sample size and patient characteristics (such as age and environment) between the two studies may be responsible for the inconsistencies in the findings. Besides zonulin, our study also evaluated the association between occludin and ADHD. Contrary to a recent study which evaluated the levels of zonulin and occludin in a smaller groups of ADHD and controls (around 40 participants in each group) [52], we did not find an association between these two gut leakage markers and ADHD in our enlarged sample. Our negative association may serve as an important reference for further studies regarding the association between occludin and ADHD. Interestingly, we found a positive correlation between occludin levels and detectability in the CPT, indicating that occludin may be associated with inattentiveness and the ability to differentiate targets from nontargets. Occludin serves as an integral membrane (transmembrane) protein of the tight junction barrier and contributes to the blood–brain barrier. Although not correlated with cognitive function, Cakir et al. [52] also reported a positive correlation between occludin levels and Conners’ parent rating scale scores, which is related to clinical symptoms. On the other hand, the level of occludin has been found to be elevated corresponding to inflammation in human postmortem brain tissue in schizophrenia [53]. Based on the above information, we propose that increased occludin may be associated with the clinical presentation and attention deficit of ADHD through the mechanism of inflammation. However, future studies are required to clarify how occludin is involved in the pathophysiology of cognitive impairment in ADHD.

Although we found a correlation between the CPT and occludin, we did not find any correlation between the gut leakage markers with the other subtest of the CPT besides detectability or any of the subtests in CATA. CPT3 generally detects visual attention while CATA examines auditory attention. A developmental lag in the maturation of the brain may lead to deficits in visual and auditory attention in ADHD [54,55]. The auditory and visual attention systems may have different developmental trajectories [56] and be associated with different networks in the brain area. The frontal and parietal network may be associated with the underlying mechanism of visual attention [57]. The temporal network and frontal network may be responsible for selective auditory attention [58]. We propose that the gut leakage markers may be more influential in the brain areas related to visual hallucination rather than auditory hallucination in patients with ADHD—possibly certain frontal and parietal areas. Previous studies reported that the performance of the CPT may be associated with the value of fractional anisotropy of the genu of the corpus callosum and left forceps minor shown in diffusion tensor imaging [59]. It was also reported that microbiota may pose an important role in enhancing the development of white matter in early life [60]. Although the correlation between gut leakage markers and the development of white matter in certain brain areas is still unknown, it is possible that some gut leakage markers may be involved in the development and maturation of white matter in brain areas associated with the performance of the CPT. However, further study is required to clarify whether gut leakage markers affect different brain areas differently and the mechanism behind it.

Our study has the following limitations. First, we may not establish a causal relationship with the current study design. This is a cross-sectional design study analyzing correlations rather than a longitudinal study which may follow temporal changes. In this way, our study was unable to control for many early-life factors that may influence the initial colonization and development of microbiota in the gut system. We did not control for types of birth delivery, breastfeeding or not, dietary patterns, the use of probiotics, and previous antibiotic treatment [61,62] for these factors were not available in this study. To sum up, it will be insufficient to draw a causal relationship between gut leakage markers and ADHD from the current study. Second, the gut leakage markers we measured were from serum samples but not directly from the brain. Whether our positive findings reflect the same association in the brain requires further evidence. On the other hand, we did not measure any immune markers in the current study. To elucidate the underlying mechanism of interaction between gut microbiota and the immune system, a future investigation is needed. Third, although we controlled for sex and BMI in Table 3 to analyze the effect of ADHD status on DEFA1 levels, there may be other demographic characteristics which may confound the gut leakage markers such as diet patterns that were not controlled for in the current study. Fourth, the correlation between DEFA1 and the SNAP-IV parent form (inattentiveness) and that between occludin levels and detectability in the CPT may not survive a correction for multiple comparison. Our result is still preliminary and future investigations with a larger sample size are warranted to confirm our findings. Finally, our study result may not be generalized across different ethnic groups or socioenvironmental circumstances. Our study is the first to evaluate these three gut leakage markers with both ADHD’s clinical symptoms and cognitive functions in a relatively moderate sample size. Whether our findings may be replicated in a larger sample size or applicable in other populations regarding heterogeneity in ethnic, sample size, study design, or drug-naive status still warrants further research.

In conclusion, we report significant differences in the gut leakage marker DEFA1 between the children with ADHD and the HC group in the current study. In addition, the serum level of DEFA1 showed an inverse correlation with both the inattention and hyperactivity/impulsivity scores reported by parents and teachers, reflecting its association with the clinical symptoms of ADHD. We also found a positive correlation between occludin levels and detectability in the CPT, indicating that gut leakage markers may not only be associated with vulnerability to ADHD, but also associated with its cognitive deficit, inattentiveness. Our results suggest that the gut leakage mechanism may play a significant role in the clinical presentation, cognitive deficits, and pathophysiology of ADHD.

## Figures and Tables

**Table 1:** Characteristics of patients with ADHD and healthy control children.

	ADHD (*n* = 130)	Controls (*n* = 73)	Statistical Values ^a^	*p* Value
Sex (*n*, %)			χ^2^ = 5.648	0.017 *
Boy	102 (78.5)	46 (63)		
Girl	28 (21.5)	27 (37)		
Age (y)	9.2 ± 2.1	9.3 ± 2.2	*t* = −0.290	0.772
Height (cm)	136.1 ± 14.4	136.8 ± 14.0	*t =* −0.347	0.729
Weight (kg)	36.1 ± 14.8	33.6 ± 11.6	*t =* 1.322	0.188
BMI (kg/m^2^)	18.8 ± 4.5	17.5 ± 3.5	*t =* 2.155	0.032 *
Clinical measures				
SNAP-IV parent form (I)	16.5 ± 5.5	5.7 ± 6.0	*t =* 12.921	<0.001 *
SNAP-IV parent form (H)	14.3 ± 6.5	4.5 ± 5.5	*t =* 10.916	<0.001 *
SNAP-IV teacher form (I)	14.8 ± 5.8	4.5 ± 4.9	*t =* 12.651	<0.001 *
SNAP-IV teacher form (H)	11.7 ± 6.8	2.8 ± 3.5	*t =* 10.268	<0.001 *
CPT				
Omission	58.7 ± 17.9	51.8 ± 13.8	*t =* 2.843	0.005 *
Commission	49.2 ± 9.8	46.2 ± 10.5	*t =* 2.055	0.041 *
Detectability	51.1 ± 9.0	48.2 ± 10.9	*t =* 2.082	0.039 *
CATA				
Omission	52.5 ± 9.3	48.8 ± 6.0	*t =* 3.014	0.003 *
Commission	53.7 ± 9.1	50.6 ± 7.7	*t =* 2.432	0.016 *
Detectability	55.5 ± 7.5	50.0 ± 9.1	*t =* 4.570	<0.001 *
Gut leakage markers				
Zonulin (μg/mL)	752.3 ± 635.1	816.9 ± 528.3	*t =* −0.733	0.464
Occludin (ng/mL)	0.09 ± 0.12	0.08 ± 0.08	*t =* 0.618	0.537
DEFA1 (ng/mL)	46.2 ± 6.5	50.2 ± 11.8	*t =* −2.702	0.008

Notes: Data are expressed as mean ± SD or *n* (%); SNAP-IV, the Swanson, Nolan, and Pelham–Version IV Scale for ADHD; I, inattention scores; H, hyperactivity/impulsivity scores. ^a^ Statistical values are expressed as *t*-value or χ^2^: * *p* < 0.05.

**Table 2:** Correlations between gut leakage markers and ADHD symptoms among all participants.

	Zonulin		Occludin		DEFA1	
	*r*	*p*	*r*	*p*	*r*	*p*
Clinical measures						
SNAP-IV parent form (I)	−0.035	0.632	0.016	0.826	−0.146	0.042 *
SNAP-IV parent form (H)	−0.023	0.745	0.106	0.141	−0.066	0.362
SNAP-IV teacher form (I)	−0.005	0.949	0.27	0.713	**−0.187**	0.010 *
SNAP-IV teacher form (H)	−0.001	0.989	0.048	0.516	**−0.179**	0.014 *
CPT						
Omission	0.060	0.404	0.028	0.692	−0.059	0.411
Commission	0.072	0.317	0.05	0.484	−0.072	0.316
Detectability	0.113	0.116	0.166	0.020*	−0.035	0.630
CATA						
Omission	0.006	0.928	−0.073	0.314	0.002	0.975
Commission	0.06	0.406	−0.074	0.304	−0.001	0.991
Detectability	0.075	0.300	−0.059	0.410	−0.125	0.082

Notes: Data are expressed as *r* (correlation coefficient); SNAP-IV, the Swanson, Nolan, and Pelham–Version IV Scale for ADHD; I, inattention scores; H, hyperactivity/impulsivity scores. * *p* < 0.05. If we used Bonferroni correction to adjust for multiple testing in the correlation matrix (*p*-value = 0.05/3 = 0.017), DEFA1 levels were significantly correlated with inattention and hyperactivity/impulsivity scores in SNAP-IV teacher form. Significant correlation remains after Bonferroni correction is expressed in bold-face type.

**Table 3:** The effect of ADHD status on DEFA1 levels examined using linear regression model, controlling for the potential confounding effects of sex and BMI.

	B (95% CI)	*p* Value
ADHD (controls vs. cases)	2.53 (0.19, 4.87)	0.034
Sex (boy vs. girl)	1.54 (−0.97, 4.05)	0.229
BMI (kg/m^2^)	−0.04 (−0.31, 0.24)	0.794

## Data Availability

The data supporting the findings of this study are available from the corresponding author, L.J.W., upon reasonable request.
